# The expanding global genomics landscape: Converging priorities from national genomics programs

**DOI:** 10.1016/j.ajhg.2025.02.008

**Published:** 2025-03-10

**Authors:** Caitlin Howley, Matilda A. Haas, Wadha A. Al Muftah, Robert B. Annan, Eric D. Green, Bettina Lundgren, Richard H. Scott, Zornitza Stark, Patrick Tan, Kathryn N. North, Tiffany Boughtwood

**Affiliations:** 1 Australian Genomics, Melbourne, VIC 3052, Australia; 2 Murdoch Children’s Research Institute, Melbourne, VIC 3052, Australia; 3 Qatar Genome Program, Qatar Precision Health Institute, Qatar Foundation, Doha, Qatar; 4 Department of Genetic Medicine, Weill Cornell Medicine, Doha, Qatar; 5 Genome Canada, Ottawa, ON K2P 1P1, Canada; 6 National Human Genome Research Institute, National Institutes of Health, Bethesda, MD 20892-2152, USA; 7 Danish National Genome Center, Ørestads Boulevard 5, Building 208, 2300 Copenhagen, Denmark; 8 Genomics England, One Canada Square, London E14 5AB, UK; 9 Great Ormond Street Hospital for Children NHS Foundation Trust, Great Ormond Street, London WC1N 3JH, UK; 10 UCL Great Ormond Street Institute of Child Health, 30 Guilford Street, London WC1N 1EH, UK; 11 Victorian Clinical Genetics Services, Murdoch Children’s Research Institute, Melbourne, VIC 3052, Australia; 12 University of Melbourne, Melbourne, VIC 3010, Australia; 13 Precision Health Research, Singapore 139234, Singapore; 14 SingHealth Duke-NUS Institute of Precision Medicine, Singapore 169609, Singapore; 15 Genome Institute of Singapore, Agency for Science, Technology and Research (A^∗^STAR), Singapore 138672, Singapore

**Keywords:** genetics, research translation, genomic medicine, public health genomics, genomic research investment, precision medicine

## Abstract

The global landscape of health genomics is expanding rapidly, with an increasing number of national and international initiatives, many of which are targeted toward accelerating the clinical implementation of genomic technologies and services in the context of local health systems. This includes a range of entities with different levels of maturity, funding sources, and strategies that focus on research and clinical priorities to varying degrees. While there is no “one-size-fits-all” approach, analysis of national genomics programs helps to identify common priority areas, barriers, and enablers. Here, we synthesize the converging priorities of several national genomics programs to highlight the importance of progressing genomics research and clinical implementation on a national scale.

## Graphical abstract

**Figure:**
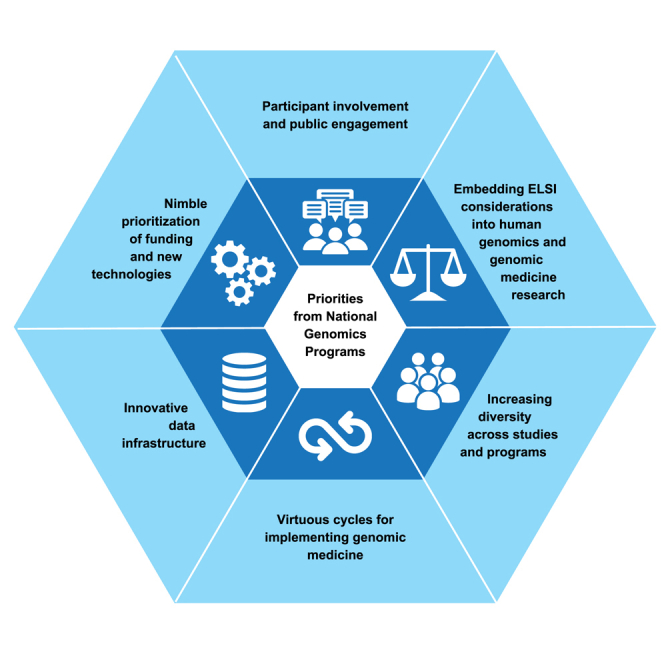

## Teaser

As the global landscape of genomics continues to expand, evaluation is needed to inform the design of current and future genomics initiatives. Six key priorities emerged from reviewing and synthesizing information on several national genomics programs. This contributes to the knowledge base and highlights a range of approaches being implemented.

## Introduction

Advances in genomic technologies are enabling unprecedented opportunities to transform the diagnosis, treatment, and management of many genetic conditions. The past two decades have seen the cost of DNA sequencing reduce by more than a million-fold, along with significant investments made toward integrating genomics into healthcare.^1^^,^^2^ A growing number of national genomics programs are providing evidence to guide the system-wide changes required for the clinical implementation of genomics research.^2^^,^^3^^,^^4^^,^^5^^,^^6^^,^^7^^,^^8^

More than 96 major genomics programs have been launched to address barriers to genomic medicine implementation across many different countries.^9^ Several large-scale national and international initiatives, including the All of Us Research Program in the United States, the European “1+ Million Genomes” Initiative, and China’s Precision Medicine Initiative, are each aiming to sequence the genomes of 1,000,000 individuals to guide evidence-based precision medicine approaches (see web resources).^10^^,^^11^

National genomics initiatives often seek to leverage capabilities and address issues that are unique to their local healthcare system; hence, there is no “one-size-fits-all” approach when it comes to the implementation of genomic medicine. Published reviews and frameworks have identified a range of complex issues, such as workforce capability and capacity, the integration and interpretation of data, public acceptability, inconsistent reimbursement, and the development of data infrastructure with robust ethical and legal frameworks.^2^^,^^4^^,^^8^^,^^12^ These reviews have also highlighted the critical role of national-level initiatives in driving the integration of genomics into healthcare. As the field matures, national genomics programs are shifting focus from the diagnostic utility of genomics in rare diseases and cancer to sustainable and equitable clinical implementation.

In this perspective, we synthesize information related to seven active national human genomics programs: Genomics England; Genome Canada; the National Human Genome Research Institute (NHGRI); Precision Health Research, Singapore (PRECISE); the Danish National Genome Center (DNGC); the Qatar Genome Program (QGP); and Australian Genomics. We have identified six priority areas shared by these programs: (1) participant involvement and public engagement; (2) embedding ethical, legal, and social implications (ELSI) considerations into human genomics and genomic medicine research; (3) increasing diversity across studies and programs; (4) virtuous cycles for implementing genomic medicine; (5) innovative data infrastructure; and (6) nimble prioritization of funding and new technologies. We highlight examples of how these priorities are being addressed to compare approaches and inform the design of new genomics programs (Table 1).^4^^,^^7^^,^^13^ Continued investment in each of these inter-related priority areas is required to drive progress toward the broad implementation of genomic medicine globally.

**Table 1:** Funding for national genomics programs with key priority areas and examples

	**Genomics England**	**Genome Canada**	**National Human Genome Research Institute (NHGRI)**	**Precision Health Research, Singapore (PRECISE)**	**Danish National Genome Center (DNGC)**	**Qatar Genome Program (QGP)**	**Australian Genomics**
Funding (average per annum)^a^	£58.4 M (USD $71.5 M)	CAD $68.6 M (USD $47.7 M)	USD $607.9 M	unavailable	DKK 162.5 M (USD $22.4 M)	unavailable	Australian Genomics: AUD $4.9 M (USD $3.0 M)
Priorities for human genomics research	genomic healthcare research and partnerships patients and participants	research and innovation genomics in society inclusive genomics capacity building, skills development, and training	structure and biology of the human genome and biology of disease genomic medicine ethical, legal, and social implications (ELSI) research data science training and workforce development small business innovation	research and precision medicine innovation and clinical implementation pilots (CIPs) enterprise and collaboration data access	patients and citizens infrastructure with equal patient access nationally ethical principles and data security integrate personalized medicine research into healthcare	research and precision health integration of genome data with electronic health record predictive genomics for disease prevention genetic testing and reporting (wellness and lifestyle) return of actionable genetic findings following American College of Medical Genetics and Genomics (ACMG) recommendations development of screening strategies and tools education and genomic literacy capacity building and skills development	nationally coordinated support for government-funded genomics research and data sharing research collaboration and building networks consolidation, communication, implementation, and informing policy
Participant involvement and public engagement	participant panel patient and participant involvement representatives ethics and engagement embedded in programs	stakeholder roundtables All for One symposia and citizen science programs	strategy development and virtual roundtables multiple working groups tied to established advisory committees outreach partnerships	citizens’ jury and public consultations for data collection and storage methods public consultations for engagement with industry	citizens’ surveys advisory board for patients, citizens, and ethics public debates	public surveys educational courses and resources gamification – Genome Heroes app	community representatives and advisory group Involve Australia networks and seminars
Clinical partnerships	National Health Service (NHS) England Genomic Medicine Service (GMS)	provincial genome centers Canadian COVID-19 Genomics Network (CanCOGeN) All for One	US academic medical centers and other healthcare organizations National Institutes of Health (NIH), including NIH Clinical Center and NIH Common Fund initiatives	PRECISE-SG100K public healthcare institutions (CIPs) regional genomics initiatives	working group for clinical use of whole-genome sequencing (WGS) specialist networks for selected patient groups sequence 60,000 genomes in healthcare by 2026	partnerships with national stakeholders under the Qatar Precision Health Institute (QPHI): Hamad Medical Corporation (HMC) Primary Health Care Corporation (PHCC) Sidra Medicine Ministry of Public Health (MOPH)	Australian Functional Genomics Network clinical flagships partnerships with state and territory clinical genetics services and genomics alliances
Priority populations	minority and underrepresented groups	Indigenous peoples	Indigenous peoples LGBTQI+ low- and middle-income countries	Singaporean population (Chinese, Indian, Malay)	Danish population	Qatari population and long-term residents (who have lived in Qatar for at least 15 years)	Indigenous peoples culturally and linguistically diverse (CALD) communities marginalized groups
Data infrastructure	national bioinformatic platform, knowledge bases, and products Research Environment: providing access to genomic, clinical, other omic, and image data	disruptive innovation and technologies data platforms and resources, including Pan-Canadian Genome Library and Health Data Ecosystem	openly available software and analysis tools data resources	Trusted Research and Real World-Data Utilization and Sharing Tech (TRUST): data sharing and linkage	National High Performance Computing Center cloud infrastructure clinical sequencing centers National Genome Database interpretation platform technical working groups	research portal bioinformatics group interpretation platform genome and multi-omics database	National Approach to Genomic Information Management (NAGIM) digital platforms, including dynamic consent platform (CTRL) and platform for sharing genomic variant interpretations (Shariant)

a Average funding figures are estimates based on publicly available financial information for each program (see web resources), noting that the granularity of financial information and definition of a financial year varied between programs.

## Participant involvement and public engagement

In 2019, a systematic review of 96 human genomics programs found that only a third (33%) reported some form of public involvement.^9^ However, participant involvement and public engagement are now regarded as essential components of genomic medicine initiatives to maximize utility and equity and ensure that genomics research is shaped by those who experience its impacts.^9^^,^^14^^,^^15^ Community engagement also aligns genomics programs with societal preferences regarding research participation, data sharing, and the investment of public funds into these programs.^16^ The lived experience of participants from diverse communities is required to address unmet needs and guide the equitable implementation of research outcomes (see web resources). Standardized frameworks increasingly facilitate the evaluation of community involvement strategies and inform best practices.^9^^,^^15^^,^^17^

Each of the national genomics programs reviewed here reports participant involvement and/or public engagement. Activities range from information provision via social media campaigns to community-led programs with involvement in policy decision-making, governance structures, and priority setting. Social media offers the widest reach for raising genomics awareness but does not always provide meaningful bi-directional communication or direct policy impacts and thus is often used to complement deliberative methods of engagement,^15^ and this is reflected in the approaches that are being utilized by national genomics programs.

Examples of public engagement strategies include Genomics England’s “Behind the Genes” podcast, Australian Genomics' “DNA Dialogue” seminars, and virtual roundtables hosted by the NHGRI (USA) to facilitate public discussions about genomics and associated ELSI topics (see web resources). Embedding ethics and engagement is exemplified by Genomics England’s public dialogue on the implications of whole-genome sequencing (WGS) for newborn screening.^18^ Outcomes from this dialogue and other public engagement activities informed the design of the Newborn Genomes Programme, which is delivering the National Health Service (NHS)-embedded Generation Study (see web resources). The QGP (Qatar) and DNGC (Denmark) have both conducted surveys to assess public attitudes toward their respective programs. The QGP has developed educational courses and resources to enhance genomic literacy across different age groups, including their “Genome Heroes” mobile application for children to learn about genomics in Arabic and/or English (see web resources).^19^

Examples of ongoing participant partnerships include the National Advisory Board for Patients, Citizens and Ethics, which advises the DNGC on the involvement of patients and citizens and ethical considerations in personalized medicine (see web resources). Genomics England’s participant panel has shaped decision-making since 2016, with members sitting on the access review, ethics advisory, and research network committees.^17^ The participant panel published recommendations for the meaningful involvement of participants in genomics research and developed a language and terminology guide for professionals in genomics (see web resources).^17^ Similarly, projects coordinated by Australian Genomics have informed the development of principles and guidelines, including for community involvement in genomics research and the management of genomic findings beyond the initial test indication (see web resources).^20^

Patient engagement in Genome Canada’s All for One initiative is helping to align genomic solutions with unmet needs in the rare disease community, with a strategy and pilot program that are guided by an advisory committee of rare disease patient advocates, clinicians, researchers, and policy experts (see web resources).

Deliberative approaches, like citizens’ juries, can empower members of the public to develop recommendations for complex ELSI that arise in genomics research while also helping to promote transparency and maintain public trust.^21^^,^^22^^,^^23^ A citizens’ jury and a workshop with religious authorities were convened during phase 1 of Singapore’s National Precision Medicine (NPM) program to explore the sharing of precision medicine data with private industry in Singapore.^24^^,^^25^ The published outcomes highlight the need for transparent decision-making and a robust governance framework to ensure data privacy. The data processes for PRECISE (Singapore) have been shaped by the outcomes of these public engagements, along with feedback from focus groups and online surveys (see web resources).

In summary, these national genomics programs are applying a variety of participant engagement and public involvement strategies to explore and address complex issues arising in human genomics research and genomic medicine implementation. Investment in a range of approaches is required to raise broad awareness of genomic advances and ensure that these advances occur in a socially acceptable manner.

## Embedding ELSI considerations into human genomics and genomic medicine research

The Human Genome Project was the first large-scale biomedical research effort with an associated ELSI component to guide responsible research conduct and outcomes.^26^ ELSI research in genomics has since grown into its own field, now regularly informing policy development and decision-making, particularly in relation to data sharing,^27^^,^^28^^,^^29^ privacy,^30^^,^^31^^,^^32^^,^^33^ genetic discrimination,^34^^,^^35^ additional (incidental) findings,^36^ and re-analysis practices.^37^^,^^38^^,^^39^

Some national genomics programs allocate a proportion of their budget to ELSI and prioritize this research through specific initiatives and by embedding it across programs. The NHGRI (USA) has a formal ELSI Research Program that began as part of the institute’s involvement in the Human Genome Project; more recently, the US Congress made it a requirement that the NHGRI spends at least 5% of the institute’s research budget on ELSI research.^1^^,^^40^ The NHGRI’s ELSI Research Program funds investigator-initiated (i.e., standalone) ELSI genomics research projects, training in ELSI research, centers of excellence in ELSI research, and one center for ELSI resources and analysis (see web resources). Genome Canada has a history of funding standalone research for genomics and its ethical, environmental, economic, legal, and social aspects (GE^3^LS), and each large-scale applied research project (LSARP) must include an integrated GE^3^LS component with a dedicated GE^3^LS lead (see web resources). Genomics England similarly embeds ethics into its programs. For example, impacts of the ethics workstream for the Newborn Genomes Programme include the development of a comprehensive consent model and changes to the language used to describe the generation study (see web resources).

The regulation and ethics workgroup from phase 1 of Singapore’s NPM provides another example of an advisory group established to identify and address ethical and regulatory barriers to the implementation of precision medicine.^41^ This workgroup developed a code of practice to regulate the provision of clinical laboratory genetic testing (see web resources).^41^ It also collaborated with the Life Insurance Association and Ministry of Health to address the need for legal frameworks that prevent genetic discrimination by issuing a moratorium on genetic testing and insurance (see web resources).^41^

In Denmark, written consent is required for comprehensive genetic analysis, and participants must specify their preferences for receiving additional (incidental) health-related findings with or without options for treatment or prevention (see web resources). Legislation was implemented to ensure that all data from genetic analysis performed after July 1, 2019, are reported to the National Genome Database hosted by the DNGC,^13^ and the DNGC Act includes a statutory purpose limitation that protects data stored by the DNGC against use in insurance or pension cases (see web resources). Information security and data protection are key focuses for the DNGC infrastructure, which became ISO certified in 2022.

ELSI considerations are inherent to the sensitive nature of genetic and genomic data and the familial implications of genomic determinants of health.^5^ National genomics programs are recognizing this and prioritizing ELSI research through standalone funding and by increasingly embedding this research within and across initiatives.

## Increasing diversity across studies and programs

The underrepresentation of diverse ancestries, minorities, and marginalized populations remains a significant barrier to achieving equitable outcomes in genomics research and genomic medicine.^16^^,^^42^ This is reflected by the pervasive bias toward European ancestry among the individuals whose genomic data reside in databases and participants studied in genome-wide association studies (GWASs).^43^ For example, European ancestry represents nearly 95% of participants in the NHGRI-European Bioinformatics Institute (NHGRI-EBI) GWAS Catalog.^44^^,^^45^ Health inequalities are further exacerbated due to the decreased effectiveness of interpreting genomic variants in the absence of diverse databases, with individuals of non-European ancestry more likely to receive inconclusive genetic test results (such as reported variants of uncertain significance [VUSs]) and false positive and false negative diagnoses.^46^^,^^47^ This underrepresentation needs to be addressed by conducting genomics research in partnership with diverse populations and Indigenous communities.^48^

The national genomics programs included in our review are funding efforts to build more representative genomic datasets and promote the inclusion of research participants from diverse populations. For example, Genome Canada is prioritizing Indigenous genomics research through the Silent Genomes initiative. This 4-year project was launched in 2018 with CAD $2.2 M (USD $1.5 M) from Genome Canada and contributions from Genome British Columbia and the Canadian Institutes of Health Research (CIHR) for a collective investment of CAD $10.4 M (USD $7.2 M), with additional funding from Illumina (see web resources). The project is establishing new processes and protocols for effective governance of biological samples and data—all through an Indigenous lens. This initiative is developing the first Indigenous background variant library for First Nations, Inuit, and Métis peoples by studying a diverse group of 1,500 First Nations people and has adopted the Indigenous ethical framework of “DNA on Loan” (see web resources).^49^^,^^50^

Australian Genomics supports Indigenous priorities in genomics, including through a partnership with the Australian Alliance for Indigenous Genomics (ALIGN). This national consortium has received AUD $5.0 M (USD $3.1 M) funding from the Australian Government, and with support from The Kids Research Institute, the Australian National University, and other partners, it has established a national Indigenous governance council and jurisdictional Indigenous governance committees, with six nodes across Australia to coordinate flagship programs (see web resources).

Genomics England’s Diverse Data initiative, with £22.0 M (USD $26.9 M) funding, aims to reduce health inequities by improving outcomes for minority communities in genomic medicine (see web resources). The major programs for 2022–2025 focus on equity in genomic medicine and research, maternal health, emerging technologies and methods, and research for sickle cell disease. A live version of the Diverse Data strategy is available on the Genomics England website to enable public input (see web resources).

In Qatar, the QGP is diversifying data by scaling up data generation. This program is the largest of its kind in the Middle East and the first WGS program in the region to study Qatari and Arab genomes, which remain underrepresented in genomic datasets (see web resources). The program has three phases, and analysis of the 6,000 genomes sequenced in the first phase revealed five non-admixed subgroups in the Qatari population and hereditary genetic marker associations for 45 clinical traits.^19^^,^^51^^,^^52^ The second phase involved sequencing over 25,000 genomes, and the third phase aims to scale up the sequencing efforts to reach up to 100,000 genome sequences at the population level.^19^

A diverse genomics workforce is required to lead genomics research that involves diverse communities with equitable research and clinical outcomes. The NHGRI has highlighted the development of a diverse workforce as “an urgent priority”^53^ and released a 10-year roadmap in 2021 to increase genomics workforce diversity (see web resources). The NHGRI has established a training, diversity, and health equity (TiDHE) office within the institute and genomic research centers at minority-serving institutions (see web resources). Genome Canada and Australian Genomics have prioritized Indigenous genomics workforce development through support for the summer internship for Indigenous peoples in Genomics Canada (SING Canada) and SING Australia (see web resources).^54^ These annual programs engage individuals from diverse Indigenous communities to enhance genomics knowledge with an Indigenous and decolonial lens. Genome Canada is expanding support for SING Canada through a new partnership that extends to at least 2029 and aims to bolster Indigenous genomics leadership in Canada (see web resources).

Sustained investment in fostering diversity in genomics research and workforce development, including Indigenous career opportunities, will be required to make genomics research accessible and ensure that it respects the values and practices of diverse communities.^55^

## Virtuous cycles for implementing genomic medicine

The typical implementation of an innovation into healthcare takes an average of 17 years.^56^ The implementation of genomic medicine continues to face many challenges, none of which can be readily addressed in isolation.^3^ Implementation research and clinical partnerships are critical drivers of the systematic change required for effective implementation and adoption and must be tailored for the unique geographical or organizational context, including health system priorities, funding models, and workforce capacity.^3^^,^^57^

The national genomics programs reviewed here are prioritizing investments to build learning healthcare systems that support a virtuous cycle between genomic medicine implementation and secondary research. This is exemplified by the “infinity loop” that NHS England and Genomics England have created through a digital infrastructure and partnership model to deliver and link clinical care and research.^58^ This intertwined process relies upon data sharing between the clinical and research environments to maximize potential public health benefits and enable secondary research with a feedback loop to clinical services.^7^ The NHS Genomic Medicine Service (GMS) Research Collaborative—which is a partnership between the NHS GMS, Genomics England, and the National Institute of Health Research (NIHR)—provides an example of a collaborative effort that is embedding genomics research in healthcare while also supporting further research and development on a national scale (see web resources). The DNGC (Denmark) has also been establishing foundations for a learning healthcare system, with the implementation of nationally coordinated infrastructure for personalized medicine in 2020 and the selection of 17 patient groups for WGS with the expectation of sequencing 60,000 genomes by January 2026.

Several national genomics programs are funding demonstration projects to develop evidence-based implementation strategies and assess the feasibility of scalable genomic medicine approaches within the unique context of their local healthcare system. PRECISE awarded up to SGD $1.5 M (USD $1.1 M) each to five 2-year clinical implementation pilots (CIPs) for applying clinical genomic tests to improve patient outcomes for breast cancer, familial hypercholesterolemia, hereditary and familial cancers, primary glomerular disease, and pharmacogenomic testing (see web resources). The CIPs are also helping to standardize clinical workflows, identify strengths and weaknesses, and develop workforce capabilities. Additional funding of up to SGD $3.0 M (USD $2.2 M) was awarded in a “competition-collaboration” model, in which shortlisted applicants worked together to identify opportunities for shared infrastructure and services across CIPs, enabling cost-effective and standardized approaches across different settings.^41^

Clinical implementation studies are also being used to establish standardized procedures for future implementation. In Qatar, the QGP has a clinical implementation project where *BRCA1/2* gene screening results are returned to participants in the population study with appropriate consent to help establish referral mechanisms between the QGP and the National Center for Cancer Care and Research (NCCCR) under Hamad Medical Corporation (HMC) and develop future implementation strategies for the return of secondary genetic findings as recommended by the American College of Medical Genetics and Genomics (ACMG).

Coordination between regional and national efforts is another common focus to ensure that genomic medicine implementation aligns with unmet needs on different scales. This is especially important in countries where healthcare delivery is a sub-national responsibility. Genome Canada coordinates national programs with provincial healthcare delivery mandates by working closely with a network of regional Genome Centers funded by the provinces. Genome Canada supports the Regional Priorities Partnership Program (RP3), which funds initiatives that aim to advance genomics research and translation capacity by pursuing regional priorities, with coordinated efforts from the Genome Centers and local partners. This CAD $20.4 M (USD $14.2 M) initiative (including co-funding) has approved funding for at least 21 projects, including an initiative that is characterizing genomic variation in the Quebec population (see web resources).

These examples highlight investments from national genomics programs to enable synergistic partnerships between genomics researchers and the clinical environment. Stepwise approaches are being applied with demonstration projects to tailor implementation strategies toward unique health system contexts and strengthen the coordination of efforts on local and national scales.

## Innovative data infrastructure

Investments in data management infrastructure and efforts to scale up genomic data sharing are key to supporting the integration of genomics into healthcare and maximizing the value of datasets in clinical and research settings.^12^^,^^59^ The integration of genomic data with routinely collected clinical data holds great promise for advancing research and clinical care yet also brings technical and ethical considerations.^59^^,^^60^ Investment in digital infrastructure must recognize the importance of adopting approaches that are standardized, interoperable, secure, and extensible, and generate data that are available for research and comparable within and between health systems.^61^ Adherence to standards is necessary but not sufficient. For example, infrastructure that supports national bioinformatics pipelines and knowledge bases has been critical for standardization in the NHS GMS and Genomics England to maximize the reusability of data and clinical and research impact.

The integration of genomic data into electronic health records (EHRs) is critical to enabling the shift toward personalized models of care.^62^ The QGP is addressing the challenge of integrating pharmacogenetic data into EHRs through the Qatar Pharmacogenetics Clinical Applications and Research Enhancement Strategies (QPGx-CARES) initiative.^63^^,^^64^ The framework for this initiative includes the development of a protocol to ensure interoperability between genetic databases and clinical decision support systems.

The reviewed national genomics programs are dedicating an increasing proportion of funds toward supporting data infrastructure, including trusted research environments (TREs), to facilitate the secure sharing and linkage of data and enable secondary research. For example, the Genomics England Research Environment was launched in 2017 as a secure cloud workspace to provide approved researchers (through the Genomics England Research Network) and pharmaceutical or biotechnology partners (through the Discovery Forum) access to deidentified participant data from the 100,000 Genomes Project and research-consented patients from the NHS GMS as well as other Genomics England research studies (see web resources). At present, the Research Environment contains more than 140,000 genome sequences associated with clinical data, other omics data, and cancer image data (see web resources).

Other examples include the federated TRE announced by the DNGC in 2022 to be established within the National High Performance Computing Center in Denmark in partnership with biotechnology company Lifebit (see web resources). Infrastructure has been a major priority for the DNGC from the beginning, with a DKK 990.0 M (USD $136.8 M) framework grant from the Novo Nordisk Foundation that included DKK 102.0 M (USD $14.1 M) upfront to invest in high-performance computing capacity. Similarly, PRECISE (Singapore) is partnering with the Trusted Research and Real World-Data Utilization and Sharing Tech (TRUST) national data exchange platform to integrate genomic data from participants in the PRECISE-SG100K study with EHRs and other real-world data with appropriate consent, facilitating secondary research and longitudinal assessment of participant outcomes.^41^

Some countries, such as Canada and Australia, face challenges related to fragmented datasets, with genomic data spread across jurisdictions. Genome Canada, alongside CIHR and other key partners, is supporting federated approaches to data sharing. This includes support for the Pan-Canadian Genome Library—a CAD $15.0 M (USD $10.4 M) initiative to share federated data through a common national library (see web resources). This initiative is partnering with the Silent Genomes initiative to include Indigenous-controlled data and is supported by other disease-specific genomic data projects, including those focusing on cancer and rare diseases (see web resources).

Australian Genomics similarly supports a federated data approach and has developed recommendations for the implementation of a National Approach to Genomic Information Management (NAGIM) and the adoption of standardized approaches for the collection, storage, and use of genomic data in Australia (see web resources). Australian Genomics is building on these recommendations by progressing toward the implementation of NAGIM in clinical and research settings, in alignment with national and jurisdictional priorities.

As the use of genomic technologies in clinical settings expands, substantial funding for data storage capacity, infrastructure, and sharing capabilities will be essential to facilitate the wider implementation of genomic medicine^57^ and, in parallel, genomics research at an increasing scale.

## Nimble prioritization of funding and new technologies

Horizon scanning and evaluation are essential to address gaps in the workforce, build evidence for emerging genomic technologies, inform genomic policy, and ultimately maximize the potential public health benefits of genomics.^57^^,^^65^^,^^66^

The need for nimble responses to local and global health issues was exemplified by the near-immediate pivot of programs, such as the NHGRI (USA), Genome Canada, and Genomics England, to invest in urgent COVID-19 studies at the start of the pandemic. For example, the Canadian COVID-19 Genomics Network (CanCOGeN) was established in 2020 by Genome Canada as a pan-Canadian collaboration for large-scale viral and human host genome sequencing to inform critical decision-making across Canada during the pandemic (see web resources). Funded by CAD $40.0 M (USD $27.8 M) from the federal government over 3 years, CanCOGeN developed the Canadian VirusSeq Data Portal, which sequenced 500,000 SARS-CoV-2 isolates, and the HostSeq Databank, which sequenced the genomes of 11,300 individuals who had been exposed to or affected by SARS-CoV-2 infection (see web resources). The QGP (Qatar) and Genomics England were part of the COVID-19 Host Genetics Initiative (COVID-19 HGI)—an international collaboration that characterized the role of host genetic determinants in SARS-CoV-2 infection and COVID-19 severity.^67^^,^^68^

National genomics programs are exploring applications of new and emerging genomic technologies. The NHGRI (USA) announced a funding opportunity to support the establishment of the Machine Learning/Artificial Intelligence (ML/AI) Tools to Advance Genomic Translational Research (MAGen) Consortium to explore the feasibility of developing ML and AI tools to enhance predictions of disease manifestations in individuals with pathogenic genomic variants (one coordinating center award with a budget of up to USD $0.8 M for 5 years and 2–4 development site awards of up to USD $1.6 M per year for 5 years; see web resources). PRECISE (Singapore) also released an opportunity for academic researchers in Singapore to access long-read DNA sequencing technologies and has a 12-month collaboration with Oxford Nanopore to sequence 10,000 genomes using PromethION 48 sequencing devices as part of the PRECISE-SG100K study (see web resources).

Genomics England launched three new initiatives in 2021, including the £26.0 M (USD $31.8 M) Cancer 2.0 initiative that is being led in partnership with the NHS (see web resources). This initiative is exploring the clinical use of long-read WGS and building a cloud-based research platform using data from participants in the Research Environment, specifically genomic and clinical data combined with digital pathology and radiology images that can be analyzed with bioinformatic and ML/AI analytical tools (see web resources).

National genomics programs are pursuing new applications that build upon previous genomics programs focused on rare diseases and cancer, notably to explore population genomic screening in healthy newborns^69^ and in adults.^41^^,^^42^^,^^70^^,^^71^^,^^72^ Agile approaches for genomics research, implementation, health technology assessment (HTA), and policy decision-making are also required to keep pace with the rapidly evolving field of genomics and ensure that national genomics programs can address future local and global needs.

## Looking ahead

Many genomics programs operate at the translational interface between research and clinical care. The overarching aim is often to establish an evidence base for embedding genomic medicine in healthcare with sustained funding. However, research and clinical implementation both require ongoing investment to support all aspects of genomic medicine. The outcomes of these investments need to continuously inform policy decisions to help realize potential benefits for patients within local healthcare systems in a timely manner.

Funding for national genomics programs is often transient and can come from multiple sources, including government, the private sector, outside of the country, or a combination of these.^7^ Fostering industry partnerships in genomics is key to driving economic growth and facilitating genomic medicine implementation, particularly by focusing on agility and innovation.^30^ However, sometimes perceived commercial interests challenge public trust, particularly in relation to concerns about data sharing with the private sector. Hence, when funding comes from multiple sources, the dual interests of participants and the economy must be carefully balanced.

Funding uncertainty hinders the ability to maintain momentum, particularly if there is a lack of certainty about long-term resource allocation to sustain implementation or infrastructure into the future.^2^ Nimble prioritization and agile strategies can be critical in this sense to ensure that genomics programs continue to progress toward realizing their organizational mission amid funding uncertainties (see web resources). Organizational structures and governance also need to evolve over time, particularly as priorities shift from research to implementation. Genomics England has expanded its programs and priorities to build upon the data, infrastructure, and impacts from the 100,000 Genomes Project (see web resources). Australia provides an example of a national genomics landscape that is actively changing, with the forthcoming establishment of Genomics Australia within the Australian Government in 2025 and commitment to ongoing funding (see web resources).

As national genomics programs mature, they increasingly look beyond their own national context to nurture collaborations and build expertise, with the aim of supporting equitable access to the benefits of genomic medicine internationally and ensuring that low- to middle-income countries and underrepresented populations are not left behind.^73^ Organizations such as the Global Alliance for Genomics and Health (GA4GH) and the Global Genomic Medicine Collaborative (G2MC) are important in building collaborative networks to advance the implementation of genomic medicine on a global scale.^74^^,^^75^ Prioritizing opportunities to share expertise and lessons learned, such as through the “Tanawwo” network (which means “diversity” in Arabic) formed by the QGP under the Qatar Precision Health Institute (QPHI) with representation from 17 low- and middle-income countries (see web resources), is important to facilitate ongoing knowledge exchange and inform the design of genomics initiatives.

## Conclusion

While genomic medicine promises to transform health systems worldwide, genomics programs inevitably vary across and within different geographical, political, and cultural contexts. Here, we have highlighted that despite these variations, several national genomics programs are converging on common key priorities of participant involvement and public engagement, embedding ELSI considerations into human genomics and genomic medicine research, increasing diversity across studies and programs, virtuous cycles for implementing genomic medicine, innovative data infrastructure, and nimble prioritization of funding and new technologies.

## Data and code availability

The information sourced for the preparation of this manuscript is available on publicly accessible websites (listed in the web resources section). The review methodology and summary datasets are available from the corresponding author upon request.

## Acknowledgments

Australian Genomics is funded by the 10.13039/501100000925National Health and Medical Research Council (grants 1113531, 2000001, and 2035846) and the Medical Research Future Fund, administered by the 10.13039/100014555Murdoch Children's Research Institute.

## Author contributions

C.H., M.A.H., and T.B. researched the literature, discussed the content, and drafted the article. All authors reviewed and edited the manuscript.

## Declaration of interests

The authors declare no competing interests.

## Web resources

ALIGN, Our Governance, https://indigenousgenomics.com.au/about-us/our-governance/
All of Us, From the All of Us CEO: Keeping Our Momentum Amidst Funding Uncertainties, https://allofus.nih.gov/news-events/announcements/all-us-ceo-keeping-our-momentum-amidst-funding-uncertainties
Australian Genomics, DNA Dialogue, https://www.youtube.com/playlist?list=PLLqz_VdF_zMNDgQELAygSKceTBLwx4321
Australian Genomics, Genomic Findings – Developing standardised approaches for genomic findings beyond the original scope of the test, https://www.australiangenomics.org.au/wp-content/uploads/2024/07/Genomic-Findings_Final-Report_March-2024.pdf
Australian Genomics, National Approach to Genomic Information Management (NAGIM) Implementation Recommendations, https://www.australiangenomics.org.au/wp-content/uploads/2021/06/NAGIM-Implementation-Recommendations_January-2023.pdf
Australian Genomics, “Precision health and the genomics ecosystem in Canada” featuring Dr. Rob Annan, DNA Dialogue August 2022,” https://youtu.be/FcMev-aMzQc?si=x1NOhwOqvOphOrxU
BC Children’s Hospital Research Institute, Building an Indigenous Background Variant Library (IBVL) in Canada (Activity 3), https://www.bcchr.ca/silent-genomes-project/ibvl
BC Children’s Hospital Research Institute, Silent Genomes Project, https://www.bcchr.ca/silent-genomes-project
CIHR, Government of Canada invests $15M in first-of-its-kind Pan-Canadian Genome Library, https://www.canada.ca/en/institutes-health-research/news/2023/10/government-of-canada-invests-15m-in-first-of-its-kind-pan-canadian-genome-library.html
Danish Ministry of Health, National Strategy for Personalised Medicine 2021–2022, https://www.eng.ngc.dk/Media/637614364621421665/Danish%20Strategy%20for%20personalised%20medicine%202021%202022.pdf
Department of Health and Aged Care, Australian Government, Establishing Genomics Australia, https://www.health.gov.au/ministers/the-hon-mark-butler-mp/media/establishing-genomics-australia
Department of Health and Social Care, UK Government, Genome UK: 2022 to 2025 implementation plan for England, https://www.gov.uk/government/publications/genome-uk-2022-to-2025-implementation-plan-for-england
Department of Health and Social Care, UK Government, Over £175 million for cutting-edge genomics research, https://www.gov.uk/government/news/over-175-million-for-cutting-edge-genomics-research
DNA Today, #286 Qatar Genome Program with Dr. Said Ismail, https://dnapodcast.com/episodes/2024/4/26/286-qatar-genome-program-with-dr-said-ismail
DNGC, Annual Report 2023, https://www.ngc.dk/Media/638493695691274048/NGC%20%C3%85rsrapport%202023%20WEB%20(1).pdf
DNGC, Comprehensive genetic analysis – patient information, https://www.ngc.dk/Media/638441911379820785/Patient%20information%20concerning%20coprehensive%20genetic%20analysis.pdf
European “1+ Million Genomes” Initiative, https://digital-strategy.ec.europa.eu/en/policies/1-million-genomes
Genome Canada, Annual Report 2017-18, https://genomecanada.ca/wp-content/uploads/2023/03/GC-Annual-Report-2017-18-FINAL-EN-Revised-in-2023.pdf
Genome Canada, Annual Report 2018-19, https://genomecanada.ca/wp-content/uploads/2022/06/Genome-Canada-Annual-Report-2018-19.pdf
Genome Canada, Annual Report 2019-20. https://genomecanada.ca/wp-content/uploads/2021/12/2019-20-Annual-Report.pdf
Genome Canada, Annual Report 2020-21, https://genomecanada.ca/wp-content/uploads/2021/12/2020-21-Annual-Report.pdf
Genome Canada, Annual Report 2021-22, https://genomecanada.ca/wp-content/uploads/2022/08/GC-AnnualReport-2021-22_EN_Web-Accessible.pdf
Genome Canada, Annual Report 2022-23, https://genomecanada.ca/wp-content/uploads/2023/08/GC-AnnualReport-2022-23_EN_web.pdf
Genome Canada, CanCOGeN, https://genomecanada.ca/challenge-areas/cancogen/
Genome Canada, CanCOGeN Update July 2023, https://genomecanada.ca/wp-content/uploads/2023/07/GC-CanCOGeN_update_July-27-ACCESSIBLE-EN-.pdf
Genome Canada, CHEO and SickKids join forces to lead the way in data sharing, https://genomecanada.ca/cheo-and-sickkids-join-forces-to-lead-the-way-in-data-sharingchanging-the-course-of-rare-diseases-copy/
Genome Canada, Corporate Plan 2023-24, https://genomecanada.ca/wp-content/uploads/2023/02/Genome-Canada-Corporate-Plan-2023-24-EN-Accessible-Version.pdf
Genome Canada, Development of a reference genome representative of the population of Quebec, https://genomecanada.ca/project/development-reference-genome-representative-population-quebec/
Genome Canada, Genome Canada launches national initiative to bring precision health to patients, https://genomecanada.ca/genome-canada-launches-national-initiative-bring-precision-health-patients/
Genome Canada, Genome Canada and SING Canada partner to address the underrepresentation of Indigenous peoples in genomics, https://genomecanada.ca/events/sing-canada-partnership/
Genomics England, Annual Report 2021, https://files.genomicsengland.co.uk/documents/Genomics-England-Annual-Report-2020-2021.pdf
Genomics England, Annual Report 2022, https://files.genomicsengland.co.uk/documents/GenomicsEngland_AnnualReport_2022.pdf
Genomics England, Data in the Research Environment, https://re-docs.genomicsengland.co.uk/data_overview/
Genomics England, Diverse Data, https://www.genomicsengland.co.uk/initiatives/diverse-data
Genomics England, Diverse Data Initiative at Genomics England: Our Strategy 2022–2025, https://docs.google.com/document/d/1lE-aGWYHQWXoYoN0YBzacANdzis6ZguaFjKT5iRhXVA/edit
Genomics England, Genomics England’s GeCIP virtual Research Environment comes online, https://www.genomicsengland.co.uk/news/gecip-research-begins
Genomics England, Join the Research Network, https://www.genomicsengland.co.uk/join-us
Genomics England, Language and Terminology Guide, https://files.genomicsengland.co.uk/documents/Genomics-England-Language-Guide.pdf
Genomics England, Launching a global standard resource for cancer research, https://www.genomicsengland.co.uk/blog/launching-a-global-standard-resource-for-cancer-research
Genomics England, Newborn Genomes Programme – Ethics, https://www.genomicsengland.co.uk/initiatives/newborns/ethics?chapter=ethics-working-group-members
Genomics England, Origins, https://www.genomicsengland.co.uk/about-us/origins
Genomics England, Podcasts https://www.genomicsengland.co.uk/podcasts
Genomics England, Statement of capital following an allotment of shares on 30 April 2024, https://find-and-update.company-information.service.gov.uk/company/08493132/filing-history/MzQyMDg1NTI5MWFkaXF6a2N4/document?format=pdf&download=0
Genomics England, The Research Environment, https://www.genomicsengland.co.uk/research
Illumina, Bridging the diagnosis gap for Canada’s Indigenous children, https://sapac.illumina.com/company/news-center/feature-articles/silentgenomes.html
Involve Australia, Guidelines for Community Involvement in Genomic Research, https://www.australiangenomics.org.au/involve-australia-guidelines-for-community-involvement-in-genomic-research/
Lifebit, Danish National Genome Center Partners with Lifebit to Deliver Personalised Medicine Nationwide, https://www.lifebit.ai/blog/danish-national-genome-center-partners-lifebit
Ministry of Health, Singapore, Moratorium on Genetic Testing and Insurance, https://www.moh.gov.sg/others/resources-and-statistics/moratorium-on-genetic-testing-and-insurance
Ministry of Health, Singapore, Revised Code of Practice (2021) – Standards for The Provision of Clinical Genetic/Genomic Testing Services and Clinical Laboratory Genetic/Genomic Testing Services, https://isomer-user-content.by.gov.sg/3/897325a8-c148-4f0b-ab00-f56a12fff4f3/1-moh-cir-no-234_2020_16dec20_genetic-testing.pdf
NHGRI, Building a Diverse Genomics Workforce Action Agenda: Implementation Plan and Progress Report, https://www.genome.gov/sites/default/files/media/files/2023-05/2023_NHGRI_Diversity_Action_Agenda_Progress_Report.pdf
NHGRI, Building a Diverse Genomics Workforce: An NHGRI Action Agenda, https://www.genome.gov/sites/default/files/media/files/2021-01/NHGRI_DiversityActionAgenda.pdf
NHGRI, FY 2020 Justification of Estimates for Congressional Appropriations Committee, https://www.genome.gov/Pages/About/Budget/NHGRIFY2020CJ.pdf
NHGRI, FY 2021 Justification of Estimates for Congressional Appropriations Committee, https://www.genome.gov/sites/default/files/media/files/2020-02/NHGRIFY2021CJ.pdf
NHGRI, FY 2022 Justification of Estimates for Congressional Appropriations Committee, https://www.genome.gov/sites/default/files/media/files/2021-05/NHGRIFY2022CJ.pdf
NHGRI, FY 2023 Justification of Estimates for Congressional Appropriations Committee, https://www.genome.gov/sites/default/files/media/files/2022-03/NHGRIFY2023-Congressional-Justification.pdf
NHGRI, FY 2024 Justification of Estimates for Congressional Appropriations Committee, https://www.genome.gov/sites/default/files/media/files/2023-03/NHGRIFY2024-Congressional-Justification.pdf
NHGRI, FY 2025 Justification of Estimates for Congressional Appropriations Committee, https://www.genome.gov/sites/default/files/media/files/2024-03/NHGRIFY2025-Congressional-Justification.pdf
NHGRI, roundtable discussion: “Does genetic and genomic screening keep open the door to eugenics?”, https://www.genome.gov/event-calendar/roundtable-discussion-does-genetic-and-genomic-screening-keep-open-the-door-to-eugenics
NHGRI, roundtable discussion: “The Promise and Perils of Social and Behavioral Genomics,” https://www.genome.gov/event-calendar/roundtable-discussion-the-promise-and-perils-of-social-and-behavioral-genomics
NHMRC, 2024-25 Australian Genomics Grant Program Grant Opportunity, https://www.grants.gov.au/Go/Show?GoUuid=06259181-186f-498c-a15d-222a6e792528
NHS England, NHS Genomic Medicine Service Research Collaborative, https://www.england.nhs.uk/genomics/genomic-research/nhs-genomic-medicine-service-research-collaborative/
NIH, ML/AI Tools to Advance Genomic Translational Research (MAGen) - Coordinating Center, https://grants.nih.gov/grants/guide/rfa-files/RFA-HG-24-005.html
NIH, ML/AI Tools to Advance Genomic Translational Research (MAGen) - Development Sites, https://grants.nih.gov/grants/guide/rfa-files/RFA-HG-24-004.html
Nuffield Council on Bioethics, Sharing best practice in ethics and genomics – summary of case studies, https://cdn.nuffieldbioethics.org/wp-content/uploads/Ethics-and-genomics-summary-of-case-studies-FINAL.pdf
Oxford Nanopore, Singapore’s National Precision Medicine (NPM) Programme Engages Oxford Nanopore to Advance Understanding of the Genetics of Singapore’s Multi-Ethnic Population, https://www.businesswire.com/news/home/20240804436871/en/Singapore%E2%80%99s-National-Precision-Medicine-NPM-Programme-Engages-Oxford-Nanopore-to-Advance-Understanding-of-the-Genetics-of-Singapore%E2%80%99s-Multi-Ethnic-Population
PRECISE, Call for Sample Submission for Long Read Sequencing, https://www.npm.sg/research/call-for-sample-submission-long-read-sequencing/
PRECISE, Clinical Implementation Pilots, https://www.npm.sg/cip/
PRECISE, Singapore’s Precision Medicine Strategy – Forging RIE on a National Scale, https://www.nmrc.gov.sg/docs/default-source/default-document-library/session-6---patrick-tan.pdf?sfvrsn=1c47799b_0
PRECISE, Trusted Research and Real World Data Utilisation (TRUST), https://www.npm.sg/partners/trust/
PRECISE, Why We Do It, https://www.npm.sg/about-us/why-we-do-it/
QPHI, Genome Heroes, https://www.qphi.org.qa/genome-heroes.
QPHI, Setting up large-scale programs to empower precision health and predictive genomics, https://www.qphi.org.qa/genomic-workshop
Singapore General Hospital, PRECISE Pre-call Roadshow, https://www.sgh.com.sg/events/research/precise-pre-call-roadshow-20210726
The Kids Research Institute Australia, Powerful new network to ensure Indigenous Australians can benefit from genomic medicine, https://www.thekids.org.au/news--events/news-and-events-nav/2023/july/powerful-new-network-align/
The Terry Fox Research Institute, Digital Health and Discovery Platform, https://www.tfri.ca/our-research/digital-health-and-discovery-platform
The University of British Columbia, Paving the Road to Indigenous Genomic Equity, https://imp.med.ubc.ca/paving-the-road-to-indigenous-genomic-equity/
Think Global Health, Filling the Middle Eastern Gaps in the Human Genome, https://www.thinkglobalhealth.org/article/filling-middle-eastern-gaps-human-genome
TRUST, About Us, https://trustplatform.sg/about-us/
